# Epstein–Barr Virus Hijacks DNA Damage Response Transducers to Orchestrate Its Life Cycle

**DOI:** 10.3390/v9110341

**Published:** 2017-11-16

**Authors:** Pok Man Hau, Sai Wah Tsao

**Affiliations:** 1 Department of Anatomical and Cellular Pathology, Faculty of Medicine, The Chinese University of Hong Kong, Hong Kong, China; 2 School of Biomedical Science, Li Ka Shing Faculty of Medicine, The University of Hong Kong, Hong Kong, China; gswtsao@hku.hk

**Keywords:** Epstein–Barr virus, DNA damage response, lytic reactivation

## Abstract

The Epstein–Barr virus (EBV) is a ubiquitous virus that infects most of the human population. EBV infection is associated with multiple human cancers, including Burkitt’s lymphoma, Hodgkin’s lymphoma, a subset of gastric carcinomas, and almost all undifferentiated non-keratinizing nasopharyngeal carcinoma. Intensive research has shown that EBV triggers a DNA damage response (DDR) during primary infection and lytic reactivation. The EBV-encoded viral proteins have been implicated in deregulating the DDR signaling pathways. The consequences of DDR inactivation lead to genomic instability and promote cellular transformation. This review summarizes the current understanding of the relationship between EBV infection and the DDR transducers, including ATM (ataxia telangiectasia mutated), ATR (ATM and Rad3-related), and DNA-PK (DNA-dependent protein kinase), and discusses how EBV manipulates the DDR signaling pathways to complete the replication process of viral DNA during lytic reactivation.

## 1. Introduction

The Epstein–Barr virus (EBV), a type of human herpesvirus, is classified as a Group 1 carcinogenic agent by the International Agency for Research on Cancer (IARC). Over 90% of the human population is infected by EBV. Infection of EBV is associated with multiple human cancers, including Burkitt’s lymphoma, Hodgkin’s lymphoma, a subset of gastric carcinomas, and almost all undifferentiated (non-keratinizing) nasopharyngeal carcinoma (NPC) [1,2]. A causal role of EBV infection in B-cell lymphoma is supported by its ability to immortalize and transform B cells both in vitro and in vivo [2]. Although EBV infection is ubiquitous and has been classified as a human tumor virus, viral infection *per se* is not sufficient to initiate cancer. A causative role in nasopharyngeal carcinoma (NPC) is strongly implicated by its universal presence in NPC, particularly in the endemic regions. The incidence of NPC has a unique pattern of geographical distribution [3]. In China, NPC is commonly detected in southern regions but not in the north, albeit the prevalent EBV infection among Chinese (>90%). The consensus is that EBV is involved in NPC pathogenesis, but genetic predisposition and environmental factors are also involved [4].

The EBV is a ubiquitous virus with a 170 kilobase double-stranded DNA genome. In stably infected host cells, the EBV episomes exist in the nuclei of infected host cells as extrachromosomal DNA wrapped with histones. EBV infects both human B lymphocytes and epithelial cells. During lytic and latent infection of EBV, different sets of viral genes are expressed. Latent infection is characterized by the expression of a limited subset of viral genes essential for latency maintenance. According to the profiles of latent genes expressed, EBV latency can be subdivided into four types, namely, latency 0, I, II, and III. Except for latency 0, all latency types express the Epstein-Barr nuclear antigen 1 (EBNA1) protein, two EBV-encoded RNAs (EBER1 and EBER2), and different types and levels of EBV-encoded microRNAs. Latent membrane proteins (LMP1, LMP2A/2B) are expressed in latency types II and III, and other EBNAs (EBNA2, EBNA3 and EBNA-LP) are expressed in latency type III [2]. During latent infection, EBV utilizes the *oriP* DNA element to replicate its episomes synchronously with the host DNA, using cellular DNA replication machineries during the S phase of the cell cycle [5].

Occasionally, lytic reactivation occurs in a small fraction of infected cells. The immediate–early (IE) EBV lytic gene, *BZLF1* (Zebra, Zta), which serves as the master switch of latent to lytic infection, is expressed in response to different stimuli. The Zta acts as both the transactivator of downstream lytic genes and as a scaffolding protein tethering other viral replication proteins to the viral replication compartment inside the host cell nuclei [6,7]. The major lytic EBV proteins induced by Zta are *BRLF1* (Replication and transcription activator, Rta) and *BRRF1* (Transcriptional activator BRRF1, Mta), which are key regulators of EBV lytic infection [8]. Earlier findings have revealed that Mta expression alone could activate the lytic cycle in epithelial cells, albeit to a lesser extent compared with Zta and Rta [9,10]. The expression of Rta and Mta cooperatively activates the BZLF1 gene promoter, forming a positive feedback loop to drive maximum expression of Zta [10]. The Zta and Rta each transactivate an individual set of lytic genes and could also act co-operatively to drive early and late lytic gene expression [11,12,13].

Lytic DNA replication initiates upon the expression of viral DNA replication proteins. Two copies of the duplicated core DNA origin element, *oriLyt*, are involved in mediating the replication of the EBV genome during lytic infection, resulting in 100-fold amplification of the viral genome [14]. A rolling circle mechanism is involved in the replication of the circularized EBV genome, resulting in a long linear DNA concatemer which will be further cleaved into individual EBV genomes and packed into the viral capsid [15].

The assembly of core viral replication proteins at the *oriLyt* is essential for the amplification of the EBV genome. The origin binding protein, Zta, together with other replication proteins including BALF5 (DNA polymerase), BMRF1 (DNA polymerase processivity factor), BBLF4 (helicase), BALF2 (the single-stranded DNA binding protein), BBLF2/3 (primase-associated factor), and BSLF1 (primase), form a large replication complex to initiate DNA replication [16]. The Rta also localizes at the viral replication compartment. However, its role on DNA replication is less defined. It is believed that Rta, together with Zta, cooperatively activate the transcription of BHLF1 early reading frame (BHLF1) which is a prerequisite for DNA replication [17]. Upon the completion of viral DNA replication, the late viral genes are expressed to mediate the processes from capsid assembly to viral DNA packaging, and eventually the release of the virus from infected host cells.

In contrast to Kaposi’s sarcoma-associated herpesvirus (KSHV), which could establish latent infection in endothelial cells, EBV infection in normal pharyngeal epithelial cells is believed to be predominantly lytic in nature and is responsible for the generation of infectious EBV for transmission [18,19]. Interestingly, EBV establishes latent infection in dysplastic and NPC lesions [20] suggesting genetic alterations in nasopharyngeal epithelial cells may support the switch of lytic to latent infection of EBV [21]. Cellular stress, including hypoxia and differentiation, could effectively turn on the virus-productive lytic infection in EBV-infected epithelial cells [22]. One of the signaling pathways commonly activated during lytic reactivation of EBV and other DNA viruses is the DNA damage response (DDR) [23,24]. Recent studies showed that both latent and lytic viral proteins are implicated during the host DNA damage response, and may contribute to cell transformation and sensitivity of infected cells to DNA damaging agents [25,26,27,28]. The discovery and functional roles of DDR transducers has been recently reviewed [29]. In this minireview, we will summarize the recent findings discerning the relationship between EBV infection and key DDR transducers including ATM (ataxia telangiectasia mutated), ATR (ATM and Rad3-related), and DNA-PK (DNA-dependent protein kinase). Moreover, we will also discuss EBV viral proteins that affect other DNA repair pathways, including translesion synthesis (TLS). We will particularly focus our discussion on how EBV manipulates the DDR signaling pathways during lytic infection to complete the viral DNA replication.

## 2. Major Transducers in DNA Damage Response (DDR)

Extensive DNA lesions occur in cells due to metabolic waste products, exposure to external physical and chemical agents including UV irradiation in sunlight, and mutagens in the environment [30]. Exposure to these mutagens results in different extents of lesion to the DNA double helix including base oxidation, bulky adducts, single-stranded break (SSB), and the more detrimental DNA lesion: the double-stranded break (DSB). The DDR signaling pathway—a surveillance mechanism to maintain genome integrity—is activated in cells to cope with these DNA lesions. The DDR is a complex signaling network that involves multiple signaling pathways to sense, transduce, and trigger effector cellular responses including cell cycle arrest, DNA repair, and apoptosis to repair the DNA damage. Cellular proteins in the family of phosphoinositide-3-kinase-related protein (PIKKs) are involved in mediating the signaling events at sites of DNA damage; these include the ATM (ataxia telangiectasia mutated), ATR (ATM and Rad3-related), and DNA-PK (DNA-dependent protein kinase), which were shown to have close interplay with EBV infection.

### 2.1. ATM: A Versatile Protein for Double-Stranded DNA Repair

**Figure 1:**
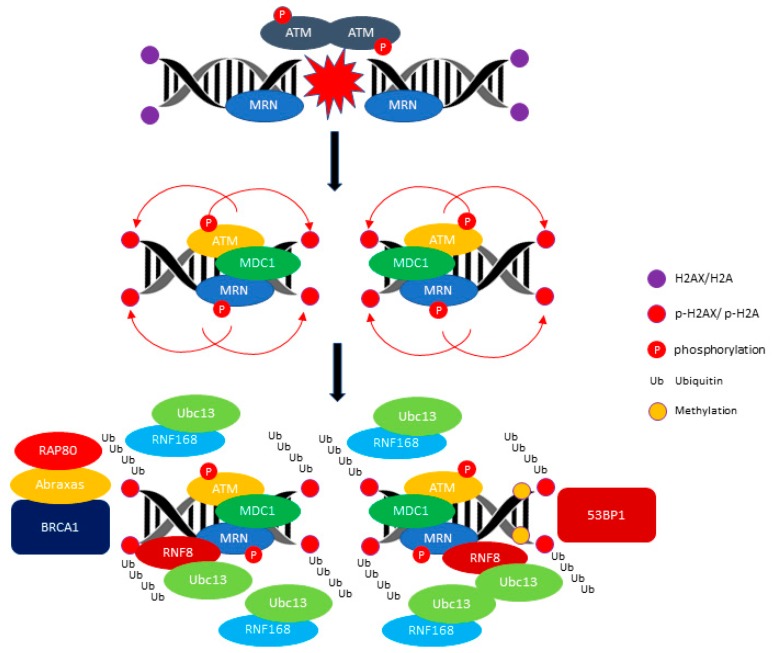
ATM (ataxia telangiectasia mutated)-mediated DDR (DNA damage response) signaling pathway. ATM phosphorylates H2AX/H2A and promotes the recruitment of E3 ubiquitin-protein ligase RNF8 (RNF8) and E3 ubiquitin-protein ligase RNF168 (RNF168) ubiquitin ligase. The RNF8 and RNF168 mediate the polyubiquitination of Histone H2A/ H2AX (H2AX/H2A) and recruit the BRCA1/RAP80/Abraxas for activation of the HR (homologous repair) pathway. The recruitment of TP53-binding protein 1 (53BP1) requires the methylation of the histone H4 Lysine 20. The red arrow indicates the phosphorylation of H1, H2A and H2AX events and the black arrow indicates the progression events take place at DNA breakage sites.

The primary sensor of DSB is a protein complex, MRE11/RAD50/NBS1 (MRN), that mediates the recruitment of ATM to the site of the double-stranded breakage [31,32,33]. The activation of ATM involves MRN/DNA stimulation which is stabilized by auto-phosphorylation at the Serine 1981 amino acid at the DNA breakage site [34,35,36]. Moreover, the full activation of ATM requires acetylation by the histone acetyltransferase. Histone acetyltransferase KAT5 (TIP60), which is an upstream regulator of the DDR pathway [37,38,39]. Activated ATM transduces the DNA damage signals by phosphorylating multiple downstream substrates responsible for amplification of the DNA damage signals (e.g., Histone H2AX (H2AX) and Mediator of DNA damage checkpoint protein 1 (MDC1), activation of cell cycle checkpoints (e.g., CHK2, p53), DNA repair (e.g., H2AX and Breast cancer type 1 susceptibility protein (BRCA1), and apoptosis (IkB and cAbl) [40,41]. One of the earliest events that occurs at the DNA-damaged sites is phosphorylation of the histone H2AX—the variant form of histone H2A which is a component of the histone octamer in nucleosomes [42]. The phosphorylated form of H2AX provides a docking site for the binding of MDC1, which acts as a scaffold for the formation of a large protein complex which amplifies the signals surrounding the DNA breakage site [43,44]. A major function of MDC1 is to promote the recruitment of two ubiquitin ligases, E3 ubiquitin-protein ligase RNF8 (RNF8) and E3 ubiquitin-protein ligase RNF168 (RNF168), to the damage site. The RNF8 and RNF168 mediate the ubiquitination of the histone H2A, histone H2AX, and histone H1 to facilitate the recruitment of downstream effector proteins, TP53-binding protein 1 (53BP1) and BRCA1 [45,46,47,48,49,50]. The 53BP1 and BRCA1 are involved in regulating the two DNA pathways to repair the double-strand break (DSB), namely the non-homologous end joining (NHEJ) and homologous repair (HR) pathway respectively [51,52]. The events involved in the ATM-mediated DDR signaling pathway are summarized in Figure 1.

### 2.2. ATR: Resolving the Problem of Replication Forks Stalling

**Figure 2:**
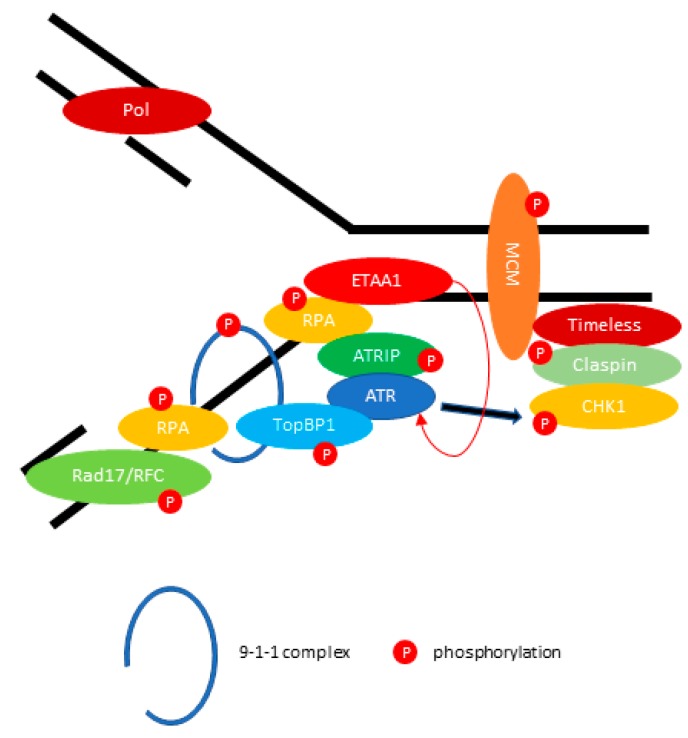
ATR (ATM and Rad3-related) mediates DNA repair during replication fork stalling. ATR kinase activity is stimulated by DNA topoisomerase 2-binding protein 1 (TopBP1) or Ewing tumor-associated antigen 1 (ETAA1). The activated ATR-ATRIP phosphorylates CHK1 through the Claspin/Timeless complex. The red arrow indicates the activation of ATR directly by ETAA1. The black arrow indicates the phosphorylation of Checkpoint kinase 1 (CHK1) through activated ATR.

Being the central coordinator for replication stress, the ATR kinase is activated in response to single-stranded DNA that occurs following the stalling of replication forks [53,54]. Replication stress during DNA replication creates stretches of exposed single-stranded DNA. These unpaired DNA strands are coated with Replication protein A (RPA) which stabilize them during DNA replication. To recruit ATR to sites of single-stranded DNA break, the ATR must first interact with its partner, ATR-interacting protein (ATRIP), which directly binds to RPA [55,56]. The RPA-coated DNA promotes the loading of the RAD17/RFC complex, which mediates the recruitment of RAD9-Checkpoint protein HUS1 (HUS1)-RAD1 (9-1-1 complex) onto DNA [57]. The subunit of the 9-1-1 complex, Rad9, interacts with the DNA topoisomerase 2-binding protein 1 (TopBP1) and stimulates the kinase activity of ATR [58,59,60]. Recently, an independent and parallel ATR activation pathway has been identified. The Ewing tumor-associated antigen 1 (ETAA1) interacts with RPA and activates ATR kinase activity independently of TopBP1 in response to replication stress [61,62,63,64]. The ETAA1-deficient cells are hypersensitive to replication stress, and loss of ETAA1 and TopBP1 leads to synthetic lethality and abrogation of ATR signaling [63]. Subsequently, activated ATR phosphorylates the effector molecule, Checkpoint kinase-1 (CHK1), through Claspin and the Timeless (Tim/Tipin) complex [65,66,67]. The ATR/CHK1 pathways are essential in protecting the genome against replication stress, and coordinating different pathways to slow down replication upon detecting DNA lesions by inhibition of replication firing, replication fork stabilization, and prevention of cell cycle progression. The events involved in ATR mediation of DNA repair during replication fork stalling are summarized in Figure 2.

### 2.3. DNA-PK: Another DSB Transducer for NHEJ DNA Repair

As aforementioned, DSB repair can be carried out by HR and NHEJ and, to a lesser extent, by MMEJ (Microhomology-mediated end joining) and SSA (Single-strand annealing) [67,68,69]. While HR functions only in the S and G2 phases of the cell cycle using homologous chromatids, NHEJ does not require the presence of a homologous counterpart and functions throughout the cell cycle, and hence is believed to be the major pathway involved in DSB repair [70,71]. The DNA ends of DSB are rapidly bound by the Ku70/Ku80 heterodimer which recruits DNA-PK catalytic subunits to DNA ends. Subsequent processing of DNA ends of DSB is carried out either by the Artemis nuclease which cuts the unpaired nucleotides, or by the polymerase µ and DNA polymerase λ which gap-fill the DNA overhangs. After the end-processing of DNA, the DNA-PK then recruits the ligation complex consisting of X-ray repair cross-complementing protein 4 (XRCC4), Xrcc4-like factor 1 (XLF), and DNA ligase IV to join the DNA strands together [72].

## 3. ATM-Mediated DDR in Response to EBV Infection

An earlier report has shown that EBV deregulated ATM during latent infection in EBV-associated Hodgkin’s lymphoma [73]. The Bmi-1, a Polycomb group protein, is up-regulated in Hodgkin’s lymphoma (HL) cells by the EBV latent membrane protein-1 (LMP1). Moreover, both Bmi-1 and LMP1 were shown to be involved in the down-regulation of ATM expression [73]. Similarly, using a panel of transfected sublines of the B-lymphoma line (BJAB) expressing the LMP1 gene, DNA repair was suppressed in LMP1-expressing cells through downregulation of the ATM [74]. Furthermore, biopsies from patients with EBV-positive undifferentiated NPC revealed down-regulation of ATM transcript and ATM protein levels [75]. In vitro infection of immortalized nasopharyngeal epithelial cell lines with a recombinant EBV was also followed by the down-regulation of ATM, and only the EBV-infected cells showed a defective DNA damage response following γ irradiation [75]. On the other hand, the LMP1 was reported to upregulate ATM in NPC cells through the activation of the NF-κB pathway [76]. The apparent discrepancy of LMP1 action on ATM expression is unclear but may be due to the different levels of LMP1 expressed in cells and different cell lines used in the investigations. Different expression levels of LMP1 may have different effects on cell fates in different cell types [77]. High levels of LMP1 often induced apoptosis in cells regardless of its well-established function in cell transformation [78]. A physiological level of LMP1 will be more relevant in interpretation of the effects of LMP1 on ATM expression.

The EBV nuclear antigen 3C (EBNA3C), which is essential for the growth transformation and immortalization of primary B lymphocytes in vitro, has been reported to disrupt the G2/M cell cycle checkpoint [79] . The EBNA3C-expressing lymphoblastoid cell lines failed to be arrested in the G2/M-phase following nocodazole treatment. Furthermore, EBNA3C interacts with the Checkpoint kinase 2 (CHK2), an effector of the ATM/ATR pathway, which is responsible for the release of G2/M arrest after nocodazole treatment. Another study revealed that activation of the ATM pathway was observed following primary infection of EBV in human B cells [28]. Interestingly, inhibition of ATM and/or CHK2 markedly increased the transformation efficiency of primary B cells. The EBV latent protein EBNA3C was required to attenuate the EBV-induced DDR to promote hyperproliferation and immortalization of primary B cells.

In summary, EBV infection disrupts ATM-mediated DDR through downregulation of ATM expression and inhibits its downstream effectors. This may contribute to genomic instability and contribute to tumorigenesis in EBV-infected cells.

## 4. EBV Disrupts the ATR-Mediated Checkpoint Response

The ATR-mediated DDR is involved in an intra-S-phase checkpoint to halt cell cycle progression upon detection of replication stress [80]. It is also involved in the control of G2/M transition [81,82]. Despite its role in regulating the cell cycle checkpoint, the ATR is also a signaling molecule that crosstalks with other signaling pathways to mediate other cellular responses. Recently, the crosstalk between the ATR-dependent DDR pathway and the Signal transducer and activator of transcription 3 (STAT3)-mediated signaling pathways has been implicated in the regulation of EBV infection [83]. Constitutive activation of STAT3 is commonly observed in EBV-infected cells, which contributes to tumor cell proliferation [84,85,86]. Recent findings have implicated STAT3 as important regulators of DDR and S-phase arrest following replication stress [87,88,89]. These findings support the hypothesis that STAT3 may enroll in DNA damage response elicited by viral infection. Another report showed that EBV infection was rapidly followed by activation and increased expression of STAT3 [90]. This resulted in relaxation of the intra-S phase checkpoint and facilitated cell proliferation. Although the replication stress-associated DNA damage is detected by the DDR, the ATR signaling in EBV infection is impaired by STAT3 activation. The ATR-CHK1 signaling could be interrupted by STAT3 through loss of Claspin, which assists ATR phosphorylation of CHK1. In contrast to the involvement of the ATR-CHK1 axis in regulating the intra-S phase checkpoint, a recent study showed that primary tonsillar B-cells infected with the prototype EBV B95.8 showed that the ATR, but not the CHK1, was critical in determining the transformation efficiency of primary tonsillar B-cells [91]. The discrepancy between the two studies may be due to different cell models and the specific EBV type used. The mechanisms involved in STAT3 expression during early infection in primary tonsillar B-cells remains to be further elucidated. As the ATR kinase phosphorylates multiple downstream targets apart from CHK1, the specific targets governing the efficiency of B cell transformations await determination. In a recent study of EBV-mediated B cell immortalization, the abrupt replicative stress resulting from hyper-proliferation was shown to activate the ATR-CHK1 pathway through depletion of nucleotide pools [92]. Interestingly, supplementation of nucleotides before hyper-proliferation of EBV-infected B cells overcame the replication stress and promoted their immortalization. These findings suggest that biogenesis of nucleotides is a critical cellular condition for the EBV transformation of B cells. The ATR-CHK1 pathway may function by closely monitoring the nucleotide pools and counteracting the virus infection by arresting cell proliferation. Thus, ATR is a key molecular switch that EBV targets to overcome the checkpoint arrest during transformation.

## 5. DNA-PK: An Emerging DDR Protein to Regulate EBV Infection

Compared with the other two DDR transducers, the involvement of DNA-PK in EBV infection has been less investigated. As an outline of the potential role that DNA-PK may have on EBV infection, the functional roles of DNA-PK on other herpesvirus subtypes are reviewed here.

Herpes simplex virus type 1 (HSV-1) attenuates DNA-PK activity by depleting the p350/DNA-PK catalytic subunit [93]. The depletion of the catalytic subunit is dependent on expression of the viral immediate–early protein, ICP0. The RNA polymerase II transcription was shown to be affected by the inhibition of DNA-PK activity. DNA affinity purification and mass spectrometry were used to identify the cellular proteins that bound to the lytic replication origins (*ori-Lyt*) of Kaposi’s sarcoma-associated herpesvirus (KSHV) [94]. The DNA-PK was found to be present at the replication initiation complexes on *ori-Lyt*. This finding implicates the involvement of DNA-PK in lytic DNA replication of KSHV. Moreover, DNA-PK phosphorylates LANA, a functional ortholog of EBNA1, negatively regulating latent viral DNA replication in KSHV [95]. Immune responses are inherent cellular properties against invading microbes. The DNA-PK has been identified as a DNA sensor that activates innate immunity [96]. The DNA-PK binds to cytoplasmic DNA and activates the transcription of type I interferon and other cytokine and chemokine genes. Cytokine response to DNA and DNA viruses is attenuated following DNA-PK depletion. In a screening of cellular proteins that interacted with EBNA-LP—the latent protein that is expressed in latency III—DNA-PK was found to interact with EBNA-LP [97]. Due to the lack of ability to recognize specific DNA sequences, the EBNA-LP relies on binding to EBNA2 to provide DNA element-specific transcriptional effects [98]. It is conceivable that DNA-PK may regulate EBNA2 transcriptional activity by interacting with EBNA2 though EBNA-LP. Recently, the viral oncoprotein LMP1 has been implicated in repressing DNA repair by inhibiting DNA-PK activity [99]. This suggests that DNA-PK is a direct target that is interrupted by EBV infection. Given that DNA-PK regulates multiple signaling pathways in other herpesviruses, it may be a critical EBV target during infection as well. More studies are warranted to reveal the involvement of this DDR regulator in EBV infection.

## 6. Other DNA Repair Pathways Affected by EBV Lytic Reactivation

Translesion synthesis (TLS) is a kind of DNA damage tolerance pathway that allows the cells to bypass DNA lesion to continue DNA replication [100]. Proliferating cell nuclear antigen (PCNA), which acts as a processivity factor for DNA polymerase and is essential for replication and DNA repair, has been revealed to be central to TLS [101]. The PCNA is monoubiquitinated in response to DNA damage and stalling replication fork. The monoubiquitinated form of PCNA recruits the polymerase η (Polη), a specialized repair polymerase involved in TLS. PCNA is implicated in EBV lytic replication of DNA as a result of its localization to the replication compartment during lytic EBV reactivation [102]. Recently, the EBV-encoded deubiquitinating (DUB) enzyme, large tegument protein deneddylase (BPLF1), has been implicated in regulating DDR. The BPLF1 is a tegument protein which is conserved across the herpesvirus family [103]. It is a late EBV gene expressed during lytic reactivation and is packed into the EBV virion. It is speculated that BPLF1 is involved in lytic reactivation through its interaction with PCNA. BPLF1 might target ubiquitinated PCNA and disrupt TLS. Interestingly, Pagano’s group has reported that overexpression of BPLF1 resulted in deubiquitination of PCNA, which abolished the localization of Polη to the nuclear foci of DNA damage and sensitized the host cells to UV and hydroxyurea treatment [104]. In addition, Rad18, the ubiquitin ligase responsible for PCNA monoubiquitination, was stabilized and relocalized to the nuclear foci of DNA replication by BPLF1 [105]. The stabilization of Rad18 was important for EBV lytic reactivation as cells infected with the BPLF1-knockout virus mutant decreased the level of Rad18 and reduced the production of the infectious virus which could be rescued by overexpressing Rad18. Furthermore, the Polη was also shown to be stabilized by BPLF1 and localized to nuclear foci similar to the recruitment of Polη to sites of DNA damage [106]. The Polη was shown to localize to EBV DNA and the knockdown of Polη resulted in decreased production of the infectious virus. These results suggested that the BPLF1 targets TLS and, as a result, might allow the cells to bypass the DNA damaged response elicited during viral DNA replication.

## 7. DDR Activation in Response to EBV Lytic DNA Replication

Extensive studies have demonstrated the activation of the DDR pathway during lytic EBV reactivation induced by different stimuli, including chemical inducers (histone deacetylase inhibitors, phorbol esters and proteasome inhibitors) and human IgG crosslinking, though the degrees to which the DDR response varies among different EBV-infected cell models [107,108]. Earlier findings on lytic EBV replication in B cells by Tsurumi’s group revealed the involvement of DDR proteins in the lytic replication of EBV DNA [109]. Using the tetracycline-inducible BZLF1-expressing B cell lines, induction of lytic EBV replication triggers a strong DNA damage response which is mediated by ATM. The DDR proteins, including activated ATM and the MRN subunits (Nbs1 and Mre11), were observed to localize at the viral replication compartment. Interestingly, the phosphorylated form of p53 (p53 Serine 15), which is ATM-dependent, interacts with the BZLF1 protein and localizes to the replication compartment, suggesting the involvement of p53 in regulating EBV gene expression during lytic DNA replication [109]. Subsequent studies also reported the expression of p53 and its stabilization during early lytic reactivation may be involved in upregulation of viral lytic gene expression (e.g., BZLF1 and BMRF1) to increase the efficiency of virus production [110,111,112,113]. The involvement of p53 activity on EBV lytic reactivation is likely to be complex. Phosphorylated p53 was shown to be degraded during the later stage of lytic reactivation [114,115]. The degradation of p53 is facilitated by the interaction of the BZLF1 protein and the Elongin B/C-Cul2/5-SOCS-box protein ubiquitin ligase complex (ECS). The BZLF1 protein may function as an adaptor for ECS and promote the ubiquitination of p53 for degradation. Degradation of p53 was suggested to promote an S-phase environment which is preferable for viral replication [113].

Using a high throughput human protein microarray, the acetyl-transferase TIP60, an upstream regulator of ATM, was shown to be required for efficient viral replication [116]. The Serine/threonine-protein kinase BGLF4 (BGLF4), an EBV-encoded protein kinase, and its orthologs in other herpesvirus families could phosphorylate TIP60 and promote its enzymatic activity. The activated TIP60 then acetylates ATM and promotes efficient viral DNA replication. The use of a specific ATM inhibitor (KU-55933) resulted in a reduction in virus production. However, a later study reported that ATM activation is required for the efficient expression of early lytic genes but not directly involved in lytic DNA replication [112]. Overexpression of BZLF1 bypasses the ATM pathway on early lytic gene induction. Similar results were also observed by an independent group which showed that the KU-55933 prevented maximal lytic genes expression (BZLF1 and BMRF1) in Raji and HH514-16 cells induced by TPA [117]. It was proposed that ATM was activated at the Zp promoter to induce maximal expression of BZLF1 and BMRF1 [117]. We also reported that lytic gene expression was suppressed by KU-55933 in EBV-infected epithelial cells induced to undergo lytic infection by TPA and sodium butyrate [118]. These observations imply that ATM activation is required for maximal induction of BZLF1 during early lytic reactivation. These findings support a role of ATM in the regulation of lytic gene expression in EBV.

**Figure 3:**
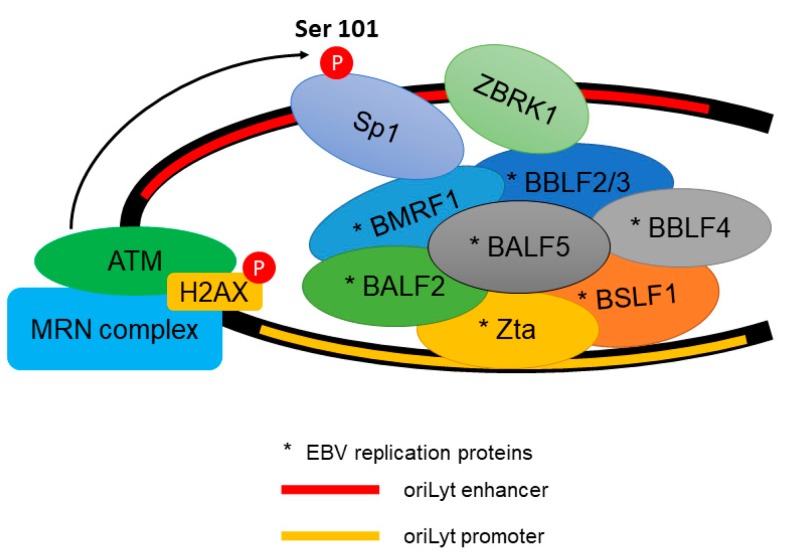
The recruitment of EBV (Epstein–Barr virus) lytic replication proteins to the oriLyt of the EBV genome. ATM phosphorylates Transcription factor Sp1 (Sp1) at Serine 101 residue. The phosphorylated Sp1 tethers and stabilizes other replication proteins which promote viral DNA replication.

In view of the localization of activated ATM at the replication compartment of EBV in the nucleus, we have investigated the functional roles of activated ATM at these discrete sites. Knockdown of ATM in EBV-infected epithelial cells resulted in the loss of the replication compartment despite abundant expression of viral early lytic proteins (BZLF1, BMRF1, BALF2, BALF5, BALF4, BSLH1 and BBLF2/3) which are responsible for lytic DNA replication [118]. This has led us to investigate the role of activated ATM in the formation of the viral replication compartment. An earlier study has shown the interaction of both viral and host proteins at the *oriLyt* [119]. We further showed that Transcription factor Sp1 (Sp1) was phosphorylated by ATM in response to DNA damage and accumulated at DNA damaged sites [120,121]. Moreover, Sp1 was reported to play an important role in mediating DSB repair independent of its sequence binding specificity [122]. Interestingly, Sp1 could be hyper-phosphorylated by ATM in response to an HSV-1 infection [123]. We reported that Sp1 phosphorylation may be an essential event downstream of ATM activation, mediating the formation of the viral replication compartment [118]. Phosphorylation of Sp1 at Serine 101 by ATM was recruited to the replication compartment during EBV lytic reactivation. We further showed that the phosphorylated Sp1, but not the unphosphorylated form, tethered the viral DNA replication proteins to the replication compartment. Our results support a role of ATM in the formation of the replication compartment in addition to its role in the regulation of expression of viral lytic genes (Figure 3).

## 8. Summary

**Figure 4:**
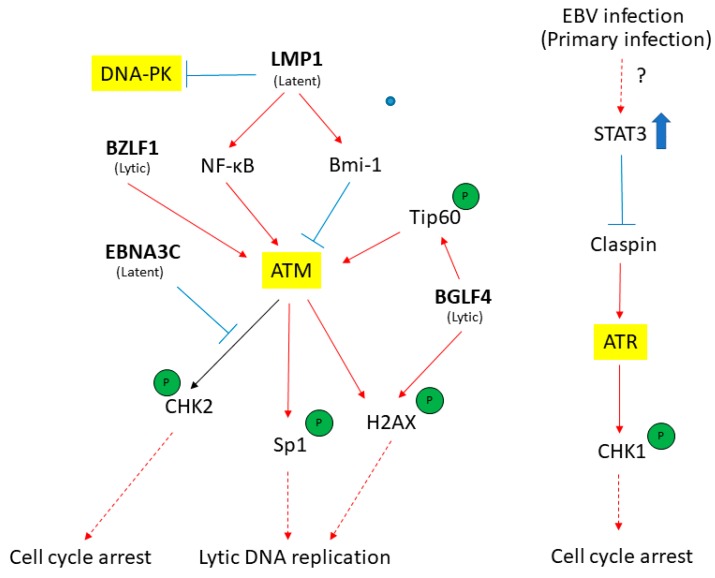
The EBV latent and lytic genes deregulates DDR transducers in EBV-infected cells. A simplified version of signaling pathways is shown here. Viral components (in bold) that activate (red) or suppress (blue) DDR transducers are shown. See texts for detail description. T-bar indicates inhibition effect. The red arrow indicates the activation of proteins. The blue rough arrow shows the increase in STAT3 activation.

In this review, we have summarized the recent findings on the involvement of major DDR transducers in the EBV life cycle (Figure 4). EBV infection and the expression of viral proteins contribute to various processes involved in cellular transformation. Given that the DDR functions as a host cell mechanism for antiviral defense as well as monitoring genome integrity, it is not surprising that the DDR pathways are targeted by EBV during the early stages of primary infection. There is evidence to support the involvement of DDR in both latency establishment and lytic reactivation of EBV. The current review focuses on the roles of DDR transducers in lytic EBV infection. Other DNA repair proteins and DDR effectors have also been reported to be involved in the regulation of the viral infection cycle, including the mismatch repair proteins and other homologous recombination (HR) proteins which have been reported to be tethered at the replication compartment for efficient viral DNA replication [102,124]. The BZLF1 was shown to interact with 53BP1 during lytic reactivation of EBV in infected cells [125]. These findings suggest that the viral DNA replication process is closely monitored by the DNA repair proteins to ensure its fidelity. However, the expression of BZLF1 may contribute to host genomic instability as shown by the activation of ATM-mediated DDR and mis-localization of DDR effector proteins following DNA damage [126]. Elucidation on the involvement of the DDR response in EBV infection may provide opportunity for therapeutic intervention in EBV-associated cancers.
